# Impact of concomitant systemic treatments on toxicity and intracerebral response after stereotactic radiotherapy for brain metastases

**DOI:** 10.1186/s12885-020-07491-z

**Published:** 2020-10-13

**Authors:** Morgan Guénolé, François Lucia, Vincent Bourbonne, Gurvan Dissaux, Emmanuelle Reygagne, Gaëlle Goasduff, Olivier Pradier, Ulrike Schick

**Affiliations:** 1 Radiation Oncology Department, University Hospital Morvan, 2 Avenue Foch, F-29200 Brest, France; 2 Latim INSERM UMR 1101, UBO, Brest, France

**Keywords:** Stereotactic radiotherapy, Brain metastases, Systemic therapies, Immunotherapy, Radioimmunotherapy

## Abstract

**Background:**

The aim of this study was to determine the safety and efficacy of fractionated stereotactic radiotherapy (SRT) in combination with systemic therapies (ST) for brain metastases (BM).

**Methods:**

Ninety-nine patients (171 BM) received SRT and concurrent ST (group 1) and 95 patients (131 BM) received SRT alone without concurrent ST (group 2). SRT was planned on a linear accelerator, using volumetric modulated arc therapy. All ST were allowed including chemotherapy (CT), immunotherapy (IT), targeted therapy (TT) and hormonotherapy (HT). Treatment was considered to be concurrent if the timing between the drug administration and SRT did not exceed 1 month. Local control (LC), freedom for distant brain metastases (FFDBM), overall survival (OS) and radionecrosis (RN) were evaluated.

**Results:**

After a median follow-up of 11.9 months (range 0.7–29.7), there was no significant difference between the two groups. However, patients who received concurrent IT (*n* = 30) had better 1-year LC, OS, FFDBM but a higher RN rate compared to patients who did not: 96% versus 78% (*p = 0.02*), 89% versus 77% (*p = 0.02*), 76% versus 53% *(p = 0.004*) and 80% versus 90% (*p = 0.03*), respectively. In multivariate analysis, concurrent IT (*p = 0.022*) and tumor volume < 2.07 cc (*p = 0.039*) were significantly correlated with improvement of LC. The addition of IT to SRT compared to SRT alone was associated with an increased risk of RN (*p* = 0.03).

**Conclusion:**

SRT delivered concurrently with IT seems to be associated with improved LC, FFDBM and OS as well as with a higher rate of RN.

## Background

Brain metastases (BM) are frequent in the natural history of several solid tumors and represent an important cause of morbidity and mortality despite active treatments [1]. For several decades, palliative whole brain radiotherapy (RT) has been the standard of care in these patients, allowing however only minor improvement in overall survival (OS) at the price of substantial neurotoxicity [2–4]. In the last decade, stereotactic radiotherapy (SRT) has emerged as a local and potentially curative treatment in a subset of patients with a limited number of BM. This approach indeed improves local control rates and reduces toxicity compared to whole brain RT [5–7].

For a long time, double-strand DNA damage in tumor cells and microenvironment were considered as the predominant mechanisms of action of RT [8]. But, recently, several studies have shown that RT, in response to antigen presentation, can induce immunomodulatory changes such as release of cytokines, increase in tumor-infiltrating lymphocytes and destruction of immunosuppressive stromal cells, allowing enhancement of immunogenic cell death [9, 10].

The wide implementation of SRT for BM management occurs in the era of new systemic treatment options such as tyrosine kinase inhibitors (TKI) and checkpoint inhibitors. By enhancing the host’s immune response against tumor cells, immune checkpoint inhibitors such as anti programmed death-1 (PD-1)/anti programmed death ligand-1 (PD-L1) and anti-cytotoxic T lymphocyte-associated antigen 4 (CTLA-4) monoclonal antibodies have revolutionized the treatment of multiple cancers including melanoma, non-small cell lung cancer and renal carcinoma [11–13]. Nevertheless, only a minority of patients achieve complete response. Additional strategies are thus necessary to improve treatment efficacy. As these drugs increase tumor cells sensitivity to radiation, the synergy between these modalities represents a new therapeutic option [14, 15].

Retrospective studies have shown SRT to be safe in combination with systemic therapies, especially in melanoma [16–18]. However, concerns exist regarding the potential toxicity given the interaction complexity between RT and systemic therapies and there is still an outstanding need for safety data on these therapeutic associations.

The aim of this study was to determine the safety and efficacy of SRT in combination with systemic therapies for intracranial metastases.

## Methods

### Patients characteristics

Patients treated with SRT for BM between 12/2014 and 06/2018 at our institution were included in this study. All primary tumors histologies were considered. Systemic treatments and SRT were suggested at multidisciplinary team meetings. Patients were offered SRT if the following criteria were met: number of BM ≤ 6, larger diameter < 40 mm according to magnetic resonance imaging (MRI), life expectancy > 3 months according to Diagnosis-Specific Graded Prognostic Assessment (DS-GPA), and non critical anatomic position. In patients who underwent SRT treatment more than once, each new lesion was considered separately.

The diagnosis was done by the MRI examination included Fluid-attenuated inversion recovery (FLAIR) and T1 weighted sequences. Extracranial disease status was defined by thoraco-abdomino-pelvic computed tomography (CT) with contrast enhancement or 18F-fluorodeoxyglucose (FDG) positron emission tomography/computed tomography (PET/CT).

All systemic treatments were allowed in this study including chemotherapy, immunotherapy, targeted therapy and hormonotherapy. Nivolumab was administrated at a dose of 3 mg/kg every 2 weeks, pembrolizumab at a dose of 2 mg/kg every 3 weeks, durvalumab at a dose 10 mg/kg every 2 weeks and ipilimumab at a dose of 3 mg/kg every 3 weeks, by intravenous injection. Targeted therapies were mainly used in non small cell lung cancers (anaplastic lymphoma kinase (ALK) inhibitor) and in renal cancer (TKI). More than 20 different chemotherapy regimens were used depending on the primary tumor and disease course. Platinum salts and taxane were the most represented CT drugs in patients with lung and breast cancer, respectively.

Treatment was considered to be concurrent if the timing between the drug administration and SRT did not exceed 1 month [19, 20]. If systemic treatment was administered before SRT, the date of SRT start was considered. On the opposite, in patients who received systemic treatment after irradiation, the date of the last SRT fraction was considered.

The cohort was divided into 2 groups: group 1 included patients treated with SRT and concurrent systemic treatment and group 2 included patients treated with SRT alone. All patients provided signed permission for the use of their clinical data for scientific purposes and Institutional Review Board approved this study.

### Treatment planning

According to different clinical and radiological parameters including BM size, presence of acute neurological symptoms, proximity to organs at risk (OAR), and critical anatomical position, total dose was prescribed at the planning target volume (PTV) periphery and ranged between 21 and 23.1 Gy in 3 fractions. Treatment plans were generated for a TrueBeamTM STX linac (Varian Medical Systems, Palo Alto, CA) equipped with standard Millennium MultiLeaf Collimator (MLC) with 120 leaves (thickness of 2.5 mm at isocenter and up to 8 cm followed by a thickness of 5 mm from 8 to 22 cm). Every patient was planned using a Flattening-Filter (FF) volumetric modulated arctherapy (VMAT) technique with 6X beams in the Pinnacle® treatment planning system (Philips, v9.10). We used two arcs from 182° to 178° for each VMAT plan. The maximum dose rate was set to 600 MU/min for 6X beams.

For patients treated until 06/2017, the prescribed dose to the PTV was a uniform dose of 3 × 7.7Gy in the periphery of the PTV. In the following patients, the planning protocol was modified and a dose gradient was created inside the PTV: the dose prescribed was 3 × 11 Gy at the isocenter with the 70% isodose line covering 99% of the PTV.

### Tumor response assessment and toxicity

Patients were assessed by brain MRI and neurological examination 6 weeks after SRT completion and every 3 months thereafter.

Radiological response on MRI was assessed by an expert neuroradiologist according to the Response Assessment for BM (RANO-BM) criteria [21]: local control (LC) was defined as the absence of new enhancing abnormality in the irradiated areas, and distant brain metastasis (DBM) as the presence of new BM or leptomeningeal enhancement outside the treated region.

Radionecrosis (RN) was assessed using contrast enhanced T1-, T2 weighted- and perfusion-MRI, even in asymptomatic patients. It was considered as the presence of central hypo-intensity and peripheral enhancement on T1-weighted post-contrast imaging, associated with edema on T2-weighted sequences and a lack of perfusion without any nodular highly vascularized area within the contrast enhanced lesion on perfusion MRI. If multiparametric MRI was inconclusive, an F-18 fluoroethyltyrosine (FET)-PET was performed. Histological confirmation of RN was not systematically required.

Systemic disease was evaluated by contrast enhanced total body CT scan, and/or 18-FDG CT-PET, depending on the primary disease.

### Statistical analysis

The general data behavior was described by the used of standard descriptive statistics. LC was defined from the SRT start to time of the local relapse. Intracranial progression was considered from the SRT start to the time of any new central nervous system progression. Overall survival (OS) was calculated from the date of diagnosis to the death or last follow-up date. Cut-off values of significant parameters (age, DS-GPA, RPA, number of BM, KPS, GTV volume and size of BM) were calculated using the receiver operating characteristic (ROC).

Univariate and multivariate Cox model was used as a method to estimate the independent association of a variable set with LC, freedom from distant brain metastases (FFDBM) and RN. All statistical analyses were performed using MedCalc Statistical Software version 15.8 (MedCalc Software bvba, Ostend, Belgium).

## Results

### Patient characteristics, radiation and systemic treatments

A total of 194 patients treated with SRT for 302 BM were included in this study: the first group (patients treated with SRT and concurrent systemic treatments) included 99 patients with a total of 171 BM. The second group (patients with SRT alone, without concurrent treatment) included 95 patients with a total of 131 BM.

Median age was 60 years. The majority of patients were male (56%), were in RPA class II (64%), had a KPS of 90–100% (70%), a DS-GPA score of 2.5–3 (53%), and a primary lung cancer (71.1%). Whole brain RT had been performed prior to SRT in 25% of patients.

Both groups were similar in terms of SRT parameters, clinical and histopathological characteristics except regarding the primary tumor (lung cancer being more represented in group 2), RPA class and the presence of extra cerebral metastases (more represented in group 1) (Table 1).

**Table 1:** Patients’ characteristics

	Groupe 1 Concomitant *N* = 99 (171 metastases)	%	Group 2 Non concomitant *N* = 95 (131 metastases)	%	Difference *p*-value
Gender					0.124
Female	49	49	36	38	
Male	50	51	59	62	
KPS median (range)	90	(60–100)	90	(60–100)	0.230
Age median (range)	61	(38–85)	60	(24–84)	0.530
Primary					
Lung	60/99	60	70/95	75	**0.004**
EGFR+	18/60	30	1/70	1.5	
ALK+	7/60	12	3/70	4	
PDL1 > 1%	26/60	43	1/70	1.5	
Breast	19/99	19	9/95	9	0.177
HER2+++	13/19	68	1/9	11	
Melanoma	13/99	13	4/95	4	0.049
GU	2/99	2	4/95	4	0.987
GI	5/99	5	8/95	8	0.461
Number of BMs (range)	2	(1–5)	2	(1–7)	0.998
Tumor size (cm)					
< 2 cm	129/171	76	88/131	67	0.109
2-3 cm	28/171	16	32/131	25	0.072
> 3 cm	14/171	8	11/131	8	0.831
Prior WBRT					
Yes	25	25	24	26	0.995
No	74	74	71	74	
DS GPA (range)	2,5	(0.5–3.5)	2,5	(0.5–3.5)	0.999
RPA					
I	18/99	18	45/95	47	**< 0.001**
II	78/99	79	47/95	50	**< 0.001**
III	3/99	3	3/95	3	0.673
ECM					
Yes	56	56	32	34	**0.002**
No	43	43	63	66	
Treatments					
CT	33/99	33	64/95	67	**< 0.001**
Before	14/33		48/64		
After	23/33		19/64		
IT	30/99	30	6/95	6	**< 0.001**
Before	15/30		4/6		
After	16/30		2/6		
TT	36/99	36	17/95	18	0.031
Before	22/36		9/17		
After	27/33		8/17		
HT	4/99	4	1/94	1	0.403
Dosimetric parameters					
HG	87/171	51	65/131	49	0.783
INH	84/171	49	66/131	51	

*Abbreviations*: *KPS* Karnofsky Performance Status, *EGFR* Epidermal Growth Factor Receptor, *ALK* Anaplastic lymphoma kinase, *PLD1* Programmed death-ligand 1, *HER2* Human Epidermal Growth Factor Receptor-2, *GU* Genito-urinary, *GI* Gastro-Intestinal, *BM* Brain metastases, *WBRT* Whole brain radiotherapy, *DS-GPA* Diagnostic-Specific Graded Prognostic Assessment, *RPA* Recursive partitioning analysis, *ECM* Extracranial metastases, *CT* Chemotherapy, *IT* Immunotherapy, *TT* Targeted therapy, *HT* Hormonotherapy*, HG* Homogeneous, *INH* Inhomogeneous

Regarding systemic therapy, more patients were treated with IT in group 1 *(p < 0.001)* and with CT in group 2 (*p < 0.001*). The median SRT dose was 23.1Gy in 3 fractions, with an inhomogenous dose prescription in 49% of patients (150/302 BM) (Table 1).

### Local control, distant brain free metastases and overall survival analysis

Median follow-up time was 11.6 months (1.4–17.3) in the first group and 12.1 months in the second group (0.7–29.7).

#### Local control

**Fig. 1:**
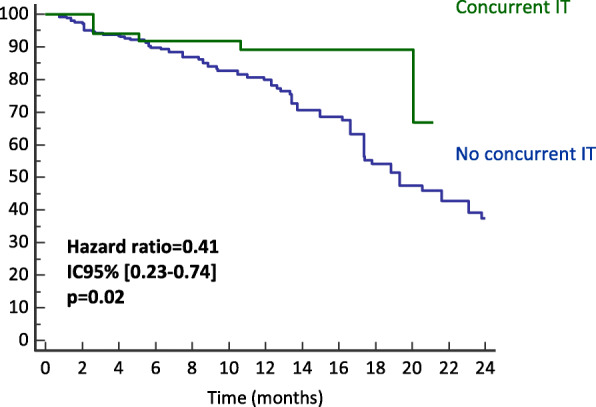
probability of Overall Survival (OS) depending on the administration of concurrent immunotherapy (IT)

Local failures occurred in 52/302 (17%) irradiated metastases. LC at 6-months and 1-year were 93% (IC95% [91–96]) and 87% (IC95% [83–91]), respectively. Twenty-seven out of 171 (15.7%) and 25/131 (19.1%) BM were not locally controlled in group 1 and 2, respectively (*p = 0.3*). On univariate analysis, factors recorded as influencing positively LC were tumor volume (< 2.07 cc), age (< 55 years), DS-GPA score (> 2.5), prior WBRT and concurrent IT. On multivariate analysis, two factors remained statistically associated with LC: tumor volume (*p = 0.039*) and concomitant IT (*p = 0.022*) (Table 2). The estimated 1-year LC rates were 96 and 78% in BM irradiated with versus without concomitant IT (*p = 0.02*) (figure 1A, supplementary materials). However, there was no impact of the treatments sequences (IT before or after RT). Similarly, 90 and 72% BM with low versus high tumor volume (cut off 2.07 cc) were still controlled at 1 year (*p = 0.03*) (figure 1B, supplementary materials).

**Table 2:** Univariate and multivariate analysis for local control (LC)

Variables	HR	Univariate analysis		OR	Multivariate analysis	
		IC95%	*p*		IC95%	*p*
Histology (lung vs. others)	0.84	0.47–1.51	0.57			
Age (≤55 vs. > 55)	1.94	2.06–3.58	**0.01**	0.98	0.95–1.01	0.11
Gender (male vs. female)	1.29	0.75–2.22	0.35			
DS-GPA (> 2.5 vs. ≤2.5)	0.49	0.27–0.88	**0.02**	0.87	0.79–1.87	0.39
RPA (1 vs. > 1)	0.73	0.41–1.28	0.29			
Number of BM (1 vs. > 1)	0.69	0.39–1.23	0.23			
ECM (yes vs. No)	1.01	0.57–1.76	0.84			
KPS (< 80% vs. ≥80%)	1.40	0.74–2.62	0.29			
**GTV volume (≥2.07 vs. < 2.07)**	**1.75**	**1.00–3.06**	**0.03**	**2.04**	**1.03–4.01**	**0.039**
Size (> 10 mm vs. < 10 mm)	1.16	0.64–2.10	0.61			
Dose distribution (HG vs. INH)	0.58	0.34–1.01	0.06			
ST concurrently (group 1 vs. group 2)	0.73	0.42–1.28	0.32			
IT (yes vs. no)	0.68	0.35–1.31	0.30			
**IT concurrently (yes vs. no IT)**	**0.33**	**0.16–0.66**	**0.02**	**0.18**	**0.12–0.79**	**0.022**
TT (yes vs. no)	0.93	0.52–1.66	0.93			
TT concurrently (yes vs. no TKI)	0.92	0.50–1.67	0.79			
CT (yes vs. no)	1.22	0.71–2.09	0.46			
CT concurrently (yes vs. no CT)	0.79	0.46–1.37	0.40			
Prior WBRT (yes vs. no)	2.26	1.16–4.48	**0.003**	0.93	0.83–2.62	0.14

*Abbreviations*: *DS-GPA* Diagnostic-Specific Graded Prognostic Assessment, *RPA* Recursive partitioning analysis, *BM* Brain metastases, *ECM* Extracranial metastases, *KPS* Karnofsky Performance Status, *GTV* Gross Tumor Volume, *HG* Homogeneous, *INH* Inhomogeneous, *ST* Systemic Treatment, *IT* Immunotherapy, *TT* Targeted therapy, *CT* Chemotherapy, *WBRT* Whole brain radiotherapy

#### Distant brain free metastases survival

Brain distance failures occurred in 60/194 patients (30.9%): 27/99 patients (27.3%) in group 1 and 33/95 patients (34.7%) in group 2 (*p* = 0.3). The 1-year freedom for distant brain metastases (FFDBM) rate was 58% (IC95% [53.8–60.6]). On univariate analysis, factors influencing FFDBM were number of BM (> 1), dose distribution (homogeneous vs inhomogeneous), concurrent systemic treatment, IT administration, and concurrent IT. There was no impact of TT or CT (*p = 0.78* and *0.82* respectively) on FFDBM, even when combined with SRT. On multivariate analysis, 3 factors remained statistically associated with FFDBM (Table 3): number of BMs (*p < 0.001*), concomitant systemic treatment (*p < 0.001*) and dose distribution (*p < 0.001*). Patients who did and did not receive concurrent systemic treatment had a 1-year FFDBM rate of 62 and 51%, respectively *(p = 0.007)*. The estimated 1-year FFDBM rates with homogeneous versus inhomogeneous dose prescription were 47 and 71% (*p < 0.001*) (figure 2A, supplementary materials). Regarding the possible impact of concurrent IT, the 1-year FFDBM rate with concurrent IT versus no concurrent IT were 76 and 53% respectively (*p = 0.004*) (figure 2B, supplementary materials). This parameter tends to significance in multivariate analysis (*p = 0.08*).

**Table 3:** Univariate and multivariate analysis for freedom for distant brain metastases

Variables	HR	Univariate analysis		OR	Multivariate analysis	
		IC95%	*p*		IC95%	*p*
Histology (lung vs. others)	0.90	0.56–1.38	0.62			
Age (> 55 vs. ≤55)	1.19	0.77–1.86	0.39			
Gender (male vs. female)	1.19	0.80–1.77	0.38			
DS-GPA (> 2.5 vs. ≤2.5)	0.90	0.61–1.34	0.29			
RPA (1 vs. > 1)	0.86	0.57–1.29	0.45			
**Number of BM (> 1 vs. 1)**	**1.52**	**1.03–2.54**	**0.03**	**1.42**	**1.18–1.72**	**< 0.001**
ECM (yes vs. No)	0.99	0.67–1.48	0.99			
KPS (< 80% vs. ≥80%)	1.18	0.77–1.80	0.35			
GTV volume (< 2.07 vs. ≥2.07)	0.81	0.55–1.80	0.25			
Size (> 10 mm vs. < 10 mm)	1.12	0.73–1.70	0.58			
**Dose distribution (HG vs. INH)**	**2.09**	**1.42–3.06**	**< 0.001**	**1.53**	**1.36–1.93**	**< 0.001**
**ST concurrently (**group 1 vs. group 2**)**	**0.60**	**0.40–0.88**	**0.007**	**0.37**	**0.21–0.67**	**< 0.001**
IT (yes vs. no)	0.47	0.29–0.75	**0.001**	0.93	0.75–4.36	0.70
IT concurrently (yes vs. no IT)	0.38	0.24–0.63	**0.004**	0.77	0.68–1.12	0.08
TT (yes vs. no)	0.81	0.54–1.21	0.32			
TT concurrently (yes vs. no TKI)	1.07	0.70–1.92	0.78			
CT (yes vs. no)	1.06	0.72–1.55	0.76			
CT concurrently (yes vs. no CT)	0.96	0.65–1.41	0.82			
Prior WBRT (yes vs. no)	0.68	0.43–1.08	0.07			

*Abbreviations*: *DS-GPA* Diagnostic-Specific Graded Prognostic Assessment, *RPA* Recursive partitioning analysis, *BM* Brain metastases, *ECM* Extracranial metastases, *KPS* Karnofsky Performance Status, *GTV* Gross Tumor Volume, *HG* Homogeneous, *INH* Inhomogeneous, *ST* Systemic treatment, *IT* Immunotherapy, *TT* Targeted therapy, *CT* Chemotherapy, *WBRT* Whole brain radiotherapy

#### Overall survival

At the last follow-up, 120/194 patients were alive (62%): 66/99 patients (66.7%) in group 1 and 54/95 patients (56.8) in group 2 (*p* = 0.2). The overall 1-year OS rate was 80% (IC95% [77.6–82.9]). On univariate analysis, age ≤ 55 years, DS-GPA > 2.5, RPA class = 1, absence of extra-cranial metastasis, Karnosky performance status (KPS) ≥80%, tumor volume < 2.07 cc and concurrent IT were significantly correlated with improved survival. However, when considering concurrent systemic treatment as a whole, no impact on OS was noted. On multivariate analysis, age, RPA score, KPS and concurrent IT remained statistically associated with improvement of OS (Table 4). The estimated 1-year OS rate was 89% versus 77% in patients treated with concurrent IT compared to those who were not (*p = 0.02*) (Fig. 1).

**Table 4:** Univariate and multivariate analysis for overall survival (OS)

Variables	HR	Univariate analysis		OR	Multivariate analysis	
		IC95%	*p*		IC95%	*p*
Histology (lung vs. others)	1.52	0.97–2.38	0.09			
**Age (> 55 vs. ≤55)**	**2.05**	**1.32–3.18**	**0.004**	**1.04**	**1.09–10.7**	**0.015**
Gender (male vs. female)	1.23	0.80–1.88	0.34			
DS-GPA (> 2.5 vs. ≤2.5)	0.38	0.25–0.58	< 0.001	0.8	0.56–1.28	0.45
**RPA (1 vs. > 1)**	**0.61**	**0.41–0.93**	**0.021**	**0.46**	**0.21–0.97**	**0.040**
Number of BM (1 vs. > 1)	0.88	0.57–1.36	0.58			
ECM (yes vs. No)	1.9	1.24–3.10	< 0.001	1.70	0.77–3.75	0.19
**KPS (≥80% vs. < 80%)**	**0.29**	**0.18–0.49**	**< 0.001**	**0.97**	**0.95–0.99**	**0.040**
GTV volume (< 2.07 vs. ≥2.07)	0.60	0.39–0.93	0.015	0.93	0.85–2.58	0.16
Size (> 10 mm vs. < 10 mm)	1.32	0.83–2.11	0.25			
Dose distribution (HG vs. INH)	1.32	0.84–2.08	0.21			
ST concurrently (group 1 vs. group 2)	0.70	0.46–1.07	0.09			
IT (yes vs. no)	0.55	0.32–0.99	0.08			
**IT concurrently (yes vs. no IT)**	**0.41**	**0.23–0.74**	**0.02**	**0.22**	**0.08–0.59**	**0.003**
TT (yes vs. no)	0.79	0.51–1.23	0.32			
TT concurrently (yes vs. no TKI)	1.01	0.65–1.60	0.95			
CT (yes vs. no)	1.07	0.70–1.63	0.73			
CT concurrently (yes vs. no CT)	0.93	0.32–1.41	0.72			

*Abbreviations*: *DS-GPA* Diagnostic-Specific Graded Prognostic Assessment, *RPA* Recursive partitioning analysis, *BM* Brain metastases, *ECM* Extracranial metastases, *KPS* Karnofsky Performance Status, *GTV* Gross Tumor Volume, *HG* Homogeneous, *INH* Inhomogeneous, *ST* Systemic Treatment, *IT* Immunotherapy, *TT* Targeted therapy, *CT* Chemotherapy

### Toxicity

SRT was well tolerated, and few acute RT-related adverse events occurred: Grade 1 and 2 nausea and headaches were reported in 17 patients (9%) with no significant difference between the 2 groups (10% in group 1 and 7% in group 2, *p = 0.57*). No grade ≥ 3 acute toxicity occurred, and no visual, motor or sensory deficits were recorded during SRT.

During the follow up, RN was suspected in 19 patients (10%): 10/99 patients (10.1%) in group 1 and 9/95 (9.5%) patients in group 2 (*p* = 0.9). Histological confirmation was obtained in 11/19 patients (58%). As this procedure was performed to exclude progression, we did not consider it as grade 3 toxicity. The 1-year RN rate was 11.5% (95%IC [9.2–13.8]). On univariate analysis, a RPA score > 1 and the use of concurrent IT were statistically associated with development of RN. On multivariate analysis, the only factor correlated with the occurrence of RN was the use of concurrent IT (*p = 0.01*). The yielding 1-year freedom from RN was 80% in patients having received concurrent IT versus 90% in patients who did not (*p = 0.03*) (Table 5) (figure 3, supplementary materials).

**Table 5:** Univariate and multivariate analysis for radionecrosis (RN)

Variables	HR	Univariate analysis		OR	Multivariate analysis	
		IC95%	*p*		IC95%	*p*
Histology (lung vs. others)	1.19	0.54–2.65	0.65			
Age (> 55 vs. ≤55)	0.71	0.31–1.63	0.38			
Gender (male vs. female)	0.89	0.43–1.89	0.77			
DS-GPA (> 2.5 vs. ≤2.5)	1.23	0.57–2.66	0.57			
RPA (1 vs. > 1)	0.40	0.20–0.85	0.03	0.85	0.53–3.15	0.58
Number of BM (1 vs. > 1)	0.52	0.24–1.14	0.07			
ECM (yes vs. No)	0.88	0.41–1.89	0.75			
KPS (< 80% vs. ≥80%)	0.99	0.42–2.34	0.99			
GTV volume (< 2.07 vs. ≥2.07)	0.71	0.33–1.51	0.35			
Size (> 10 mm vs. < 10 mm)	1.32	0.59–2.91	0.51			
Dose distribution (HG vs. INH)	2.01	0.96–4.26	0.06			
ST concurrently (group 1 vs. group 2)	2.02	0.95–4.32	0.09			
IT (yes vs. no)	2.38	0.96–5.89	0.02			
**IT concurrently (yes vs. no)**	**2.23**	**0.90–5.94**	**0.03**	**3.01**	**1.30–6.94**	**0.012**
TT (yes vs. no)	1.12	0.72–1.95	0.09			
TT concurrently (yes vs. no)	2.66	1.19–5.96	0.06			
CT (yes vs. no)	0.94	0.45–1.95	0.87			
CT concurrently (yes vs. no)	0.98	0.65–2.06	0.96			
Prior WBRT (yes vs. no)	2.88	1.23–6.73	0.07			

*Abbreviations*: *DS-GPA* Diagnostic-Specific Graded Prognostic Assessment, *RPA* Recursive partitioning analysis, *BM* Brain metastases, *ECM* Extracranial metastases, *KPS* Karnofsky Performance Status, *GTV* Gross Tumor Volume, *HG* Homogeneous, *INH* Inhomogeneous, *ST* Systemic Treatment, *IT* Immunotherapy, *TT* Targeted therapy, *CT* Chemotherapy, *WBRT* Whole brain radiotherapy

## Discussion

Although series on BMs are heterogeneous in terms of primary tumors, prognosis, treatments and patients clinical characteristics, outcome in our cohort are similar to the one reported in other studies [22, 23].

We found that addition of concurrent IT to SRT was able to increase survival and provide long term control in patients with BMs from solid tumors. In our study, the estimated 1-year LC rates were 96 and 78% in BM irradiated with versus without concurrent IT (*p = 0.022*). This finding is in line with the recent metaanalyse of Petrelli et al showing that the addition of IT to RT is associated with improved OS (HR = 0.54, 95%CI 0.44–0.67; *p < 0.001*) compared to RT alone in patients treated with BM [24]. The interesting point is that RT given before or concurrently to IT seemed to provide better results than the reverse sequence [25]. Due to the small number of patients (19 received IT before RT, 17 after RT and 9 before and after), we were not able to confirm this hypothesis. One of the explanations for the synergism of that sequence specifically is the RT induced enhancement of cross presentation of antigens by dentritic cells during cancer cell death, triggering the innate immune system to activate tumor-specific T cells [9]. Moreover, RT administered before systemic treatment may improve the blood-brain barrier permeability, allowing drugs to better penetrate metastases [26].

Moreover, we observed that patients irradiated with a heterogeneous dose developed less distant brain metastases than those irradiated with a homogenous dose (47 and 71% at 1 year (*p < 0.001*). This is in line with our previous report, highlighting the importance of SRT planning [27]. An interesting finding here is the trend to a lower intracerebral relapse rate observed in patients receiving concurrent IT compared to those who did not (24% versus 47% at 1 year, *p = 0.08*). This result should however be interpreted with caution given the small number of patients. This observation may reflect the immune-mediated “abscopal effect”, defined as tumor regression at sites distant to the irradiated field [28]. Few cases of abscopal effects have been reported in the literature so far, and optimal biological and physical conditions to trigger this immune modulation are unknown. But, case reports on abscopal effects occurring with the combination of anti-CTLA4 and SRT in patients with melanoma are increasing and suggest a clinical benefit in terms of survival [28, 29].

The release of antigen for cross presentation depends on the fraction size and total dose of radiation. But, for now, no consensus exists regarding the optimal dose to trigger antigen presentation. Low-dose radiation may better stimulate immune cells and modulate the stromal microenvironment than higher dose [30]. On the opposite, Lee et al showed that a dose of 20 Gy administered in one fraction only induces T cell proliferation in lymph nodes whereas this was not observed with the delivery of the same total dose but given in 4 fractions [31].

Radiation-induced brain necrosis is a relatively uncommon but potentially severe adverse event of SRT. Its incidence varies between 2 and 30% depending on dose prescription and isodose line, dosimetric parameters [27, 32], disease characteristics [33] as well as RN diagnosis criteria [34, 35]. Minniti et al reported RN rates that were significantly different between single- and multiple-fraction SRT (20 and 8% respectively) [32]. Other authors have reported similar results, with a lower incidence of RN (3 to 11%) with dose delivery in 3–5 fractions [36, 37]. Although the risk of RN is known to increase with time, little data are available regarding the risk in longer-term survivors, as survival has generally been poor in the era prior to effective systemic therapy. In a series on 271 BM treated with single-fraction SRT, RN occurred in 25.8% of treated lesions and, in patients still alive at 2 years, an increase to 34% was observed [33].

RN could thus be more prevalent in long survivor’s patients treated with IT. Furthermore, as RT stimulates the immune response through T-cell activation, it might exacerbate RN [38]. Few studies with limited number of patients have already suggested an increases incidence of RN with the addition of IT to SRT compared to SRT alone in patients with melanoma [39–41]. But most patients in these reports were treated with the anti-CTLA-4 monoclonal antibody Ipilimumab, and OS rarely exceeded 1 year. In a recent study of Kaidar-Person et al on longer-term surviving patients with metastatic melanoma treated with anti-PD-1 therapy, the incidence of RN was estimated as 18% at 2 years [40]. We observed a RN rate of 10% in the present study, and this rate was higher in patients receiving concomitant IT. This finding has also been reported by Martin et al, who found that receipt of IT was associated with symptomatic RN (HR, 2.56; *p = .004*) in a cohort of patients with melanoma, non–small-cell lung cancer, or renal cell carcinoma, this association being especially strong in patients with melanoma. The association with PD-1 inhibition was however not statistically significant [42].

Our study has inherent bias due to its retrospective nature. First it is heterogeneous, with various tumor types and systemic drugs represented. Many variables were tested including the RPA score, although it can be considered as a confounding factor. Indeed, RPA score combines age, KPS, presence or absence of extracranial metastasis, and control of the primary tumor, which were also tested separately. The number of patients who received IT is also limited. Additionally, patients who received concomitant systemic treatment may have been selected based on their performance status or favorable disease presentation or prognosis, and will affect outcomes given the variable time at risk to develop intracranial failure and toxicity. Moreover, oncologists are usually reluctant to interrupt IT in very good responders, this representing a potential selection bias. Systemic treatments could also impact outcome, even without RT. The delay chosen (1 month) to consider systemic treatments as concomitant is also controversial, although largely used in the literature [19, 20, 43]. In a recent meta-analysis published by Lu et al, it seems that immune checkpoint inhibitor administration within 4 weeks of stereotactic radiosurgery for BMs provides a statistically significant better 12-month OS compared to administration outside that time period [43]. Regarding toxicities, only RN was reported as it usually relies on MRI and is therefore relatively easy to collect. But, in the context of systemic treatments, others toxicities are awaited like headaches, cognitive changes, bleeding, or ataxia. These are more difficult to evaluate in the retrospective setting. Finally, the prognostic impact of concurrent IT could be related to the systemic effect of IT, instead of the timing of treatment administration. Given the fact that only 6 patients received not concurrently administered IT, analyzing this subpopulation was not meaningful.

This report provides valuable insight into tolerance and efficacy of combination of IT and SRT. Future clinical trials examining the efficacy and safety of this combination and the optimal schedule of RT in terms of irradiation field, fractionation and dose will hopefully answer these questions. For now, clinicians can rely on the guidelines of the American Society for Radiation Oncology (ASTRO) on combining precision RT with molecular targeting and immunomodulatory agents [44]. Beyond clinical factor and PDL1 expression, other parameters like measure of inflammatory cytokines or tumour-specific T-cells in serum following administration of SRT could act as surrogates for the efficacy of the treatment related immune response, and provide further insight into the appropriate selection of patients.

## Conclusion

SRT delivered concurrently with IT seems to be associated with improved LC, FFDBM and OS as well as with a higher rate of RN.

## Supplementary information

**Additional file 1: Figure 1.** Probability of local control depending on: (A) administration of concomitant immunotherapy (IT) and (B) metastases volume. **Figure 2.** Probability of freedom from distant brain metastases (FFDBM) depending on (A) Dose prescription modality and (B) Administration of concurrent immunotherapy (IT). **Figure 3.** Probability of occurrence of radionecrosis (RN) depending on the administration of concurrent immunotherapy (IT).

## Data Availability

The datasets used and/or analysed during the current study are available from the corresponding author on reasonable request.

## Abbreviations

ALK: Anaplastic lymphoma kinase
ASTRO: American Society for Radiation Oncology
BM: Brain metastases
CT: Computed tomography
CTLA-4: Cytotoxic T lymphocyte-associated antigen 4
DBM: Distant brain metastasis
DS-GPA: Diagnosis-Specific Graded Prognostic Assessment
FDG: Fluorodeoxyglucose
FET: Fluoroethyltyrosine
FF: Flattening-filter
FFDBM: Freedom from distant brain metastases
FLAIR: Fluid-attenuated inversion recovery
HT: Hormonotherapy
IT: Immunotherapy
KPS: Karnosky performance status
LC: Local control
MLC: MultiLeaf Collimator
MRI: Magnetic resonance imaging
OAR: Organs at risk
OS: Overall survival
PD-1: Programmed death-1
PD-L1: Programmed death ligand-1
PET/CT: Positron emission tomography/computed tomography
PTV: Planning target volume
RANO-BM: Response Assessment for BM
RN: Radionecrosis
ROC: Receiver operating characteristic
RT: Radiotherapy
SRT: Stereotactic radiotherapy
TKI: Tyrosine kinase inhibitors
TT: Targeted therapy
VMAT: Volumetric modulated arctherapy
