# Supplementary material for: Impact of concomitant systemic treatments on toxicity and intracerebral response after stereotactic radiotherapy for brain metastases
Source: BMC Cancer. 2020 Oct 13;20:991. doi: 10.1186/s12885-020-07491-z (PMC7557085; doi:10.1186/s12885-020-07491-z)
Supplement: Supplementary file 1 — Additional file 1: Figure 1. Probability of local control depending on: (A) administration of concomitant immunotherapy (IT) and (B) metastases volume. Figure 2. Probability of freedom from distant brain metastases (FFDBM) depending on (A) Dose prescription modality and (B) Administration of concurrent immunotherapy (IT). Figure 3. Probability of occurrence of radionecrosis (RN) depending on the administration of concurrent immunotherapy (IT). [file 12885_2020_7491_MOESM1_ESM.docx]

**Figure 1: Probability of local control depending on: (A) administration of concomitant immunotherapy (IT) and (B) metastases volume**

**A.**

Concurrent IT

No concurrent IT

**Hazard ratio=0.33**

**IC95% [0.16-0.66]**

**p=0.02**

**B.**

Tumor volume ≥2.07 cc

**Hazard ratio=1.75**

**IC95% [1.00-3.06]**

**p=0.03**

Tumor volume <2.07 cc

**Figure 2: Probability of freedom from distant brain metastases (FFDBM) depending on (A) Dose prescription modality and (B) Administration of concurrent immunotherapy (IT)**

**A.**

Homogeneous dose

**Hazard ratio=2.09**

**IC95% [1.42-3.06]**

**P<0.001**

Inhomogeneous dose

**B.**

No concurrent IT

**Hazard ratio=0.38**

**IC95% [0.24-0.63]**

**p=0.004**

Concurrent IT

**Figure 3: Probability of occurrence of radionecrosis (RN) depending on the administration of concurrent immunotherapy (IT).**

No concurrent IT

Concurrent IT

**Hazard ratio=2.23**

**IC95% [0.90-5.94]**

**p=0.03**
